# Unveiling the storm: delirium coding trends among hospitalized Peruvian older adults

**DOI:** 10.1590/1980-5764-DN-2024-0200

**Published:** 2025-01-17

**Authors:** Anthony Renzo Apuy, Camila Sofía Badell, Oscar Josue Ponce, Eloy Francisco Ruiz, Luis Enrique Palomino

**Affiliations:** 1 Universidad Peruana de Ciencias Aplicadas, Lima, Peru.; 2 Mayo Clinic, Knowledge and Evaluation Research Unit, Rochester, MN, United States of America.; 3 University Hospitals Plymouth NHS Trust, Derriford Hospital, Plymouth, United Kingdom.; 4 Icahn School of Medicine at Mount Sinai, Brookdale Department of Geriatrics and Palliative Medicine, New York, NY, United States of America.; 5 Hospital II Clinica Geriatrica San Isidro Labrador, Lima, Peru.

Dear Editor,

Delirium is a potentially life-threatening condition that affects approximately 23% of older hospitalized patients^1^. According to the Diagnostic and Statistical Manual of Mental Disorders, fifth edition (DSM-5) and the 10^th^ revision of the International Classification of Diseases (ICD-10), delirium is a syndrome characterized by an abrupt onset and a fluctuating course, marked by impaired attention, awareness, and cognition, along with evidence of an underlying organic cause^2^. Delirium increases the risk of dementia, post-discharge institutionalization, and mortality in older patients^3^. It is estimated that hospitalized geriatric patients have a mortality rate 1.5 times higher within the year following discharge^3^.

In the United States of America, delirium rates are the highest in intensive care unit and palliative care settings (70-85%), post-operative delirium is a common complication among older adults (15-25%)^4,5^. Despite these significant numbers, delirium remains inadequately documented in medical records and is rarely coded in hospital administrative systems^5^. This could lead to an underestimation of its true impact, resulting in reduced reimbursement and fewer resources allocated for its management^5^. In England and Scotland, the coding of delirium in hospitalized patients increased from approximately 1.4 to 12.2% between 2012 to 2020^1^. To our knowledge, the current reporting practices for delirium in Peru are unknown. Therefore, we aimed to analyze the trends in delirium diagnosis coding among Peruvian patients older than 60 years old, based on all hospital discharges recorded by the Peruvian Ministry of Health (MINSA) establishments from 2002 to 2022.

MINSA is the primary provider of healthcare services in Peru, covering approximately 70% of the population^6^. We requested data on the annual hospital discharges for patients older than 60 years of age in Peru from 2000 to 2022, as well as discharges coded as F05 (delirium not induced by alcohol or other psychoactive substances) according to the ICD-10. This data is publicly available from MINSA and can be formally requested at: https://www.minsa.gob.pe/portada/transparencia/solicitud/frmformulario.asp. To ensure comparability with data from the United Kingdom (UK), our methodology follows that of Ibitoye et al^1^. The prevalence of delirium was calculated by dividing the total number of patients with discharge code F05 by the total number of hospital discharges for each age group in each year.

Over the 20-year period, there were 1,406,976 hospital discharges with 309 cases of delirium recorded, resulting in a delirium prevalence consistently below 0.01% per year (Table 1). Despite this low prevalence, there was an upward trend in the number of cases, which remained consistent across age groups for both females and males. The highest number of discharged patients diagnosed with delirium was observed in the older age groups (>70 years old), with two to eight times more cases in the >80 group. Our findings reveal an exceptionally low prevalence of delirium coding among hospitalized Peruvian patients over a two-decade ­period (2002 to 2022), with a consistent rate of less than 0.01%. In contrast to trends observed in the UK^1^, where delirium prevalence increased significantly, the coding rates for delirium in older Peruvian adults approached negligible levels, suggesting substantial underdiagnosis and underreporting of this condition.

**Table 1:** Delirium prevalence rates in Peru, 2002-2022, according to age range.

Years	Ages 60-64	Ages 65-69	Ages 70-74	Ages 75-79	Ages >80
2002	0/10,440 (0.0)	1/10,124 (0.0)	1/9,158 (0.0001)	2/7,276 (0.0002)	0/11,242 (0.0)
2003	1/11,607 (0.00008)	1/11,090 (0.00009)	0/10,155 (0.0)	0/8,205 (0.0)	6/12,644 (0.0004)
2004	0/12,516 (0.0)	2/11,891 (0.0001)	3/11,149 (0.0002)	2/9,160 (0.0002)	1/13,435 (0.00007)
2005	0/13,310 (0.0)	0/12,449 (0.0)	3/11,711 (0.0002)	1/9,772 (0.0001)	4/14,834 (0.0002)
2006	0/14,977 (0.0)	0/14,300 (0.0)	0/13,430 (0.0)	0/10,883 (0.0)	1/16,767 (0.00005)
2007	0/15,258 (0.0)	0/14,728 (0.0)	2/14,135 (0.0001)	0/11,775 (0.0)	5/18,065 (0.0002)
2008	1/16,402 (0.00006)	1/15,563 (0.00006)	0/14,548 (0.0)	5/12,520 (0.0003)	8/19,096 (0.0004)
2009	0/17,847 (0.0)	1/16,635 (0.00006)	1/15,493 (0.00006)	2/13,560 (0.0001)	2/19,794 (0.0001)
2010	0/19,761 (0.0)	0/18,096 (0.0)	1/17,212 (0.00005)	1/14,948 (0.00006)	1/23,671 (0.00004)
2011	0/18,449 (0.0)	0/17,190 (0.0)	0/16,206 (0.0)	2/14,702 (0.0001)	5/22,630 (0.0002)
2012	1/18,490 (000005)	0/17,856 (0.0)	1/16,260 (0.00006)	1/14,881 (0.00006)	5/23,903 (0.0002)
2013	0/19,613 (0.0)	3/18,946 (0.0001)	0/17,371 (0.0)	0/16,049 (0.0)	4/25,256 (0.0001)
2014	0/19,457 (0.0)	2/18,145 (0.0001)	6/16,849 (0.0003)	10/15,482 (0.0006)	16/25,897 (0.0006)
2015	0/21,854 (0.0)	3/20,396 (0.0001)	5/18,578 (0.0002)	4/16,515 (0.0002)	18/26,684 (0.0006)
2016	2/20,681 (0.00009)	3/18,958 (0.0001)	1/17,259 (0.00005)	4/15,886 (0.0002)	9/25,990 (0.0003)
2017	2/20,343 (0.00009)	0/18,822 (0.0)	1/17,740 (0.00005)	4/15,875 (0.0002)	9/27,056 (0.0003)
2018	2/22,224 ((0.00008)	3/20,699 (0.0001)	2/19,220 ((0.0001)	5/16,985 ((0.0002)	17/28,449 (0.0005)
2019	3/22,802 ((0.0001)	1/21,568 (0.00004)	8/20,187 (0.0003)	8/18,008 (0.0004)	19/30,345 (0.0006)
2020	1/17,253 (0.00005)	2/15,573 (0.0001)	2/13,521 (0.0001)	2/11,165 (0.0001)	10/17,295 (0.0005)
2021	1/23,820 (0.00004)	1/21,324 (0.00004)	3/17,850 (0.0001)	6/14,483 (0.0004)	6/21,194 (0.0002)
2022	3/21,582 (0.0001)	6/20,720 (0.0002)	5/18,232 (0.0002)	7/15,848 (0.0004)	6/25,780 (0.0002)

Notes: The numerator represents the number of delirium diagnoses, while the denominator represents the total population (discharges). The number in parentheses indicates the prevalence of delirium for each age group for each year.

The increased reporting of delirium coding in the UK can be attributed to the systematic efforts within the National Health Service in the UK to improve awareness, diagnosis, and management of delirium^5^. Additionally, the availability of well-validated delirium screening tools, such as the Confusion Assessment Method (CAM) and the 4 A's test (4AT), widely used in the clinical ­diagnosis of delirium^7^, may have influenced this trend.

In Peru, MINSA has not introduced any guidelines or initiatives to raise awareness about delirium and its impact. To our knowledge, the first Spanish translation of a delirium screening tool was the Delirium Rating Scale in 1996^8^. Most recently, the CAM was validated in Spanish in 2004^8^, followed by the 4AT in 2016^9^. The delayed availability of these tools in Spanish may have hindered their integration into clinical practice in Peru, potentially contributing to the underdiagnosis of delirium. Additionally, delirium may be underreported due to discharge diagnoses are often recorded under the codes of the underlying conditions that caused it (*e.g*. infections, trauma, metabolic abnormalities) instead of using the specific code for delirium.

In conclusion, the significant discrepancies in delirium diagnosis and discharge coding indicate a potential underestimation of delirium prevalence at discharge^10^, particularly among hospitalized Peruvian older adults. Efforts should focus on enhancing healthcare worker education and implementing effective detection tools to ensure accurate recognition and reporting of ­delirium in Peru.
